# The impact of threat of shock on the framing effect and temporal discounting: executive functions unperturbed by acute stress?

**DOI:** 10.3389/fpsyg.2015.01315

**Published:** 2015-08-31

**Authors:** Oliver J. Robinson, Rebecca L. Bond, Jonathan P. Roiser

**Affiliations:** Institute of Cognitive Neuroscience, University College London, LondonUK

**Keywords:** threat of shock, stress, temporal discounting, framing effect, anxiety, depression, executive function, Bayesian models

## Abstract

Anxiety and stress-related disorders constitute a large global health burden, but are still poorly understood. Prior work has demonstrated clear impacts of stress upon basic cognitive function: biasing attention toward unexpected and potentially threatening information and instantiating a negative affective bias. However, the impact that these changes have on higher-order, executive, decision-making processes is unclear. In this study, we examined the impact of a translational within-subjects stress induction (threat of unpredictable shock) on two well-established executive decision-making biases: the framing effect (*N* = 83), and temporal discounting (*N* = 36). In both studies, we demonstrate (a) clear subjective effects of stress, and (b) clear executive decision-making biases but (c) no impact of stress on these decision-making biases. Indeed, Bayes factor analyses confirmed substantial preference for decision-making models that *did not* include stress. We posit that while stress may induce subjective mood change and alter low-level perceptual and action processes (Robinson et al., 2013c), some higher-level executive processes remain unperturbed by these impacts. As such, although stress can induce a transient affective biases and altered mood, these need not result in poor financial decision-making.

## Introduction

Stress can significantly alter the way that we perceive and react to the world, promoting the processing of threatening and unexpected information (Robinson et al., 2013b,c). This threat bias can be adaptive – improving the ability to detect and avoid further sources of stress – but this bias also likely contributes, at least in part, to the facilitatory role that stress plays in the onset of mood and anxiety disorders (Kendler et al., 2004). Threat of unpredictable shock is a reliable within-subject method of inducing stress in both humans and experimental animals (Grillon, 2008; Davis et al., 2010). While the impact of threat of shock on basic perceptual processes is relatively well studied, its impact on higher-level executive processes such as decision-making is surprisingly poorly understood (Robinson et al., 2013c). Here, we explore the impact of threat of shock on two classic decision-making biases: the framing effect and temporal discounting.

Threat of shock is a translational stress-induction procedure in which an individual anticipates an unpredictable and unpleasant electrical shock (Schmitz and Grillon, 2012). In animals threat of shock has been shown to engage neural circuitry distinct from that engaged during fear conditioning (Davis et al., 2010), another widely used but conceptually different aversive processing paradigm. More precisely, *anxiety* (or stress) is operationally defined as the prolonged apprehensive response to a context in which threats *may* occur, whereas *fear* is the acute response to a discrete, defined and *predictable* aversive stimulus or cue (Davis et al., 2010; Robinson et al., 2013c). Critically, stress induced by threat of shock has well documented psychological (Robinson et al., 2013c), psychophysiological (Grillon et al., 1991), and neural effects (Cornwell et al., 2007; Robinson et al., 2012, 2013b). Perhaps more importantly there is also emerging evidence that threat of shock evokes mechanisms related to those that participate in pathological anxiety, for example Generalized Anxiety Disorder (Robinson et al., 2013c, 2014). As such, it is hoped that exploring the impact of threat of shock on cognitive functions may also provide a window into the mechanisms which contribute to pathological stress-related disorders in healthy individuals, prior to disorder onset (Robinson et al., 2014).

Executive function is an umbrella term encompassing cognitive functions that do more than passively process information. Such non-automatic functions might integrate information from multiple sensory domains along with information stored in memory. Executive function therefore encompasses higher order processes, such as planning and decision-making. The impact of threat of shock on executive function has not been comprehensively studied. Here we explore one aspect of executive function: financial decision-making. We are aware of only two studies in which threat of shock was shown to alter financial choices. In the first, threat of shock promoted risk-avoidant decision-making (Clark et al., 2012). However, in this study, the threat cues were discrete, of short duration (5–5.5 s) and possibly more comparable to a fear cue than an anxiety/stress condition. In the second study (Robinson et al., 2015), threat of shock had no main effect on gambling choices, but did interact with trait anxiety, promoting ‘harm-avoidant’ (i.e., playing less ‘disadvantageous’ decks) under stress in those with the low anxiety symptoms. However, the Iowa gambling task used in this second study confounds a number of decision-making and basic cognitive processes, making the causes of this result unclear. In the small number of remaining studies that have addressed this question, the effects seemed to be largely restricted to reaction times (Murphy, 1959; Keinan, 1987; Engelmann et al., 2015), with stress having no impact on the decisions themselves.

Microeconomic theory has outlined a number of biases – or heuristics – which have been shown to guide individual financial decision-making behavior. In this study, we explore two well-established biases: the framing effect and temporal discounting. The framing effect describes the reliable propensity of individuals to alter their decisions, dependent on whether the same choice is ‘framed’ as a loss or a gain. Specifically, individuals tend to avoid risk when a choice is framed as a gain (e.g., keep £2 out of £10 vs. a 20% chance to win £10), and become risk-seeking when the exact same choice is framed as a loss (e.g., lose £8 out of £10 vs. a 20% chance to win £10). That is to say, all other things being equal, individuals are biased against certain outcomes ‘framed’ as losses (Kahneman and Tversky, 1979; De Martino et al., 2006). Temporal discounting is another such bias, in which an individual assigns less value to gains in the future relative to the present (Rachlin and Green, 1972; Berns et al., 2007). For instance, offered £10 today and £11 in a month, there is a bias toward accepting the lower value of £10 now. In other words, temporal distance causes devaluation of potential gains. In both paradigms, the subjective utility of financially identical options is biased by the context in which the options are presented. Given that both of these biases can be financially suboptimal and result in reduced gains/increased losses, it is plausible that they might be shifted by contexts such as stress-induced biases toward negative stimuli (Robinson et al., 2013c). Clinical support for this hypothesis comes from the observation of altered decision-making and negative biases in disorders associated with anxiety and negative affect such as major depression (Eshel and Roiser, 2010; and it should be noted that stress, negative mood, and anxiety are relatively diffuse, likely overlapping, concepts).

Therefore, we explored the impact of stress on the framing effect and temporal discounting. We predicted that threat of shock would induce a state of adaptive anxiety (Robinson et al., 2013c) and promote harm-avoidant decisions (Clark et al., 2012; Robinson et al., 2013c), thereby increasing both framing and temporal discounting. Specifically, in the context of uncertain threat an individual might be more loss averse, resulting in an increased framing effect (Porcelli and Delgado, 2009). At the same time in the context of an uncertain future, an individual might be biased toward immediate vs. future gains resulting in increased temporal discounting (Pulcu et al., 2014). This study explored these hypotheses with conventional significance testing as well as a Bayesian approach to enable a more nuanced comparison of different behavioral models.

## Materials and Methods

### Sample and Screening

Participants were recruited from the UCL Institute of Cognitive Neuroscience Subject Database, of which *N* = 83 (49 female: 34 male; mean age = 24, *SD* = 5) completed the framed gamble task and *N* = 36 (18 female: 18 male; mean age = 24, *SD* = 6) completed the temporal discounting task (*N* = 35 completed both). All participants completed a prior phone screen in which they reported no personal history of/treatment for psychiatric or neurological disorders or drug use (from a detailed specific checklist of all disorders), along with no cardiovascular problems, pacemakers, or cochlear implants. The demographics represented the naturalistic sample of individuals who responded to our call for participants and who passed screening. All subjects provided written informed consent (UCL Research Ethics Committee Project ID Number: 1764/001). Both decision tasks were presented on a desktop computer using the Cogent toolbox for Matlab (Wellcome Trust Centre for Neuroimaging and Institute of Cognitive Neuroscience, UCL, London, UK). To incentivise performance subjects were informed that additional compensation would be provided based upon task performance. Shocks were delivered to the non-dominant wrist using a DS7 stimulator (Digitimer Ltd, Welwyn Garden City, UK). Prior to testing, all subjects completed a shock work-up procedure in which shocks were titrated (over approximately 3–5 stimulations) to a level that was ‘unpleasant but not painful’ (Schmitz and Grillon, 2012).

### Anxiety Measures

At the end of each block, participants indicated how anxious they had felt during each of the threat and safe conditions on a scale from 1 (“not at all”) to 10 (“very much so”) as a subjective manipulation check. Participants also provided self-report measures of depression (Beck Depression Inventory: BDI; Beck and Steer, 1987) and trait anxiety (State Trait Anxiety Inventory: STAI; Spielberger et al., 1970) at the end of the session.

### Framed Gamble Task

**FIGURE 1:**
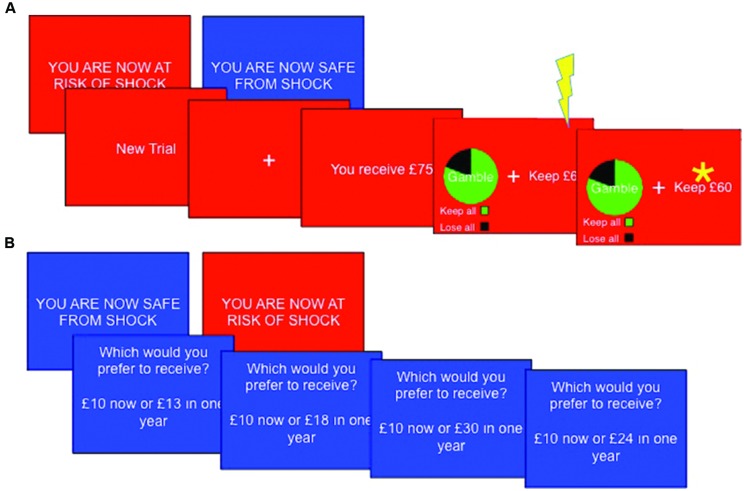
**Task sequences. (A)** The Framed gamble task (n.b asterisk represents subject choice, not outcome) and **(B)** temporal discounting task Subjects completed both tasks under threat (red background) and safe (blue background) conditions, with a total of four shocks per task occurring at a pseudorandom point within threat blocks only.

This task was adapted from that used by De Martino et al. (2006). The task consisted of eight blocks (four safe, four threat), each comprising 14 trials. “YOU ARE NOW SAFE FROM SHOCK” or “YOU ARE NOW AT RISK OF SHOCK” was presented for 3 s at the beginning of each new block. Threat blocks had a red background, whilst safe blocks had a blue background. A single shock was delivered at a pseudorandom time in each threat block. Each trial began with a message “You receive £X” where, X = varying monetary amounts. Participants then had 4 s to choose between a certain option, which would leave them with a guaranteed portion of the total £X, or an option to gamble, which could lead to either winning the entire amount or winning nothing. The participant did not discover the outcome of any gambles, but was instructed to consider which option they would choose to maximize wins and minimize losses. In gain frames the participant would have the ‘sure’ option to “Keep £Y,” a certain portion of the total whereas in loss frames participants were told they would “Lose £Z” (where Z + Y = X), implying that they would retain the rest of the total (i.e., £Y) – note that this represents precisely the same decision. Alternatively, participants could choose a gamble option which was presented with a pie chart indicating the probability of each keeping or losing the entire £X amount. In experimental trials, the expected values (sum of all possible outcomes weighted by their respective probabilities) of the gamble and sure options were matched (**Figure 1A**). Expected outcomes were 20, 40, 60, or 80% of the initial total £X, which was set as £25, £50, £75, or £100. Monetary parameters were counterbalanced across decision frames and between threat and safe blocks. ‘Catch’ trials were also included to verify that the participants were attending to and had understood the task. These trials were designed such that the expected outcome of one option was much larger than that of the other option, such that participants should always choose the option of higher value.

Each block consisted of 10 standard trials (five in each frame) and with four catch trials (one in each combination of frame and preferred option) in a random order, with the certain and gamble options randomized to the left or right of the screen. Choices were indicated by pressing the left or right arrow key. The chosen option was highlighted by a star for 1 s. Analysis was conducted on the proportion of trials on which participants chose to gamble, and reaction times to choose each of the gamble and the certain options. Before the main task, six practice trials (one of each type of catch trial, plus a standard trial in each the gains and losses frames) were completed without a time limit or threat of shock to ensure participants understood the task. Task duration was approximately 15 min.

### Temporal Discounting Task

On each trial subjects were presented with a (self-paced) choice between an immediate reward (e.g., “£5.00 now”) and a delayed reward (e.g., “£10.00 in 25 years”). The value of the delayed reward was fixed whilst the immediate reward was adjusted based upon previous choices until an indifference point was reached. Adjustment was based on Yi et al. (2010) abbreviation of Johnson and Bickel’s (2002) algorithm. Indifference points were recorded for three delayed values (£10, £100, and £1000), three delays (1 day, 1 year, and 25 years) and for both gain and loss frames (“Which would you prefer to receive?” or “Which would you prefer to lose?”), yielding a total of 18 indifference points (**Figure 1B**). The task consisted of six blocks (three safe, three threat), during each of which six (randomly selected without replacement) indifference points were reached. As the algorithm adjusted the choices presented based upon previous responses, the number of trials per block varied (range ∼60–80). The screen displayed “YOU ARE NOW SAFE FROM SHOCK” with a blue background at the start of safe blocks or “YOU ARE NOW AT RISK OF SHOCK” with a red background at the start of threat blocks. Shocks (*N* = 4) were presented in a pseudo-random order during threat blocks. Task duration was approximately 15 min.

### Analysis

Conventional frequentist significance tests were run in SPSS version 22 (IBM Corp, Armonk, NY) whilst Bayesian analyses were run in JASP, employing the default prior (Rouder et al., 2012; Love et al., 2015; Morey and Rouder, 2015). Frequentist and Bayesian repeated-measures analysis of variance (ANOVA) models were constructed in exactly the same manner for all analyses (see below), with frequentist ANOVAs used to generate F-statistics, p-values and effect sizes for interactions of interest, and Bayesian ANOVAs used to generate log Bayes factors (logBF_10_)^1^ for models of interest relative to a null model (main effect of subject).

In our Bayesian analyses, the ‘winning’ model was defined as the model with the highest BF_10_ relative to the null, and the relative predictive success of one model over another was computed by dividing the BF_10_ for one model by the other. Any value greater than zero indicates a model *better* than the comparison. Semantic labels were assigned to the magnitude of these comparisons to aid interpretation, ranging from anecdotal (1–3), to substantial (3–10), to strong (10–30) to decisive (>100; Jeffreys, 1998). Where reported for interactions, the Bayes factors represent a model including the interaction plus the main effect of each component of the interaction.

#### Statistical Models

*Manipulation efficacy* was assessed using a paired *t*-test (and Bayesian equivalent) to compare retrospective ratings across stress conditions.

For the *framed gamble task*, the proportion of trials on which participants chose the gamble option was assessed using a repeated-measures ANOVA with stress condition (threat/safe) and decision frame (gains/losses domains) as within-subject factors. Reaction time was analyzed in a similar model, with the addition of choice (gamble/sure) as an additional within-subjects factor.

For the *temporal discounting task*, we analyzed normalized indifference points (indifference point/fixed delayed value) in a repeated-measures ANOVA with stress condition (threat/safe), decision valence (gain/lose), delayed value (£10/£100/£1000) and time (1 day, 1 year, 25 years) as within-subjects factors. Reaction times for choices across each indifference point (mean reaction time of all responses required to reach indifference) were analyzed in a separate model with the same factors.

Finally, for both tasks, additional exploratory between-subjects analyses were also run including measures of mood symptoms and order of threat-safe counterbalancing as covariates. These were separate models, run after the *a priori* within-subject models. BDI symptom data was not normally distributed, and was square-root transformed prior to analysis. Further exploratory analyses suggested during peer review were also run: gender, age, and threat potentiated (threat minus safe) subjective ratings were included as additional between subject factors.

## Results

Data for these tasks are freely available for download^2^.

### Manipulation Check

Subjects reported feeling significantly more anxious in the stress relative to the safe condition in both the framed gamble [a mean rating (±*SD*) of 6 ± 2/10 relative to 2 ± 1/10; *t*(82) = –16, *p* < 0.001] and temporal discounting tasks [6 ± 2/10 relative to 2 ± 1/10; *t*(34) = –10, *p* < 0.001]. Bayes factors indicated that models including stress conditions were decisively better than the null model for both the framed gamble (logBF_10_ = 57) and temporal discounting tasks (logBF_10_ = 21).

### Framed Gamble Task

#### (A) Within-Subjects Effects

##### Choice behavior

**FIGURE 2:**
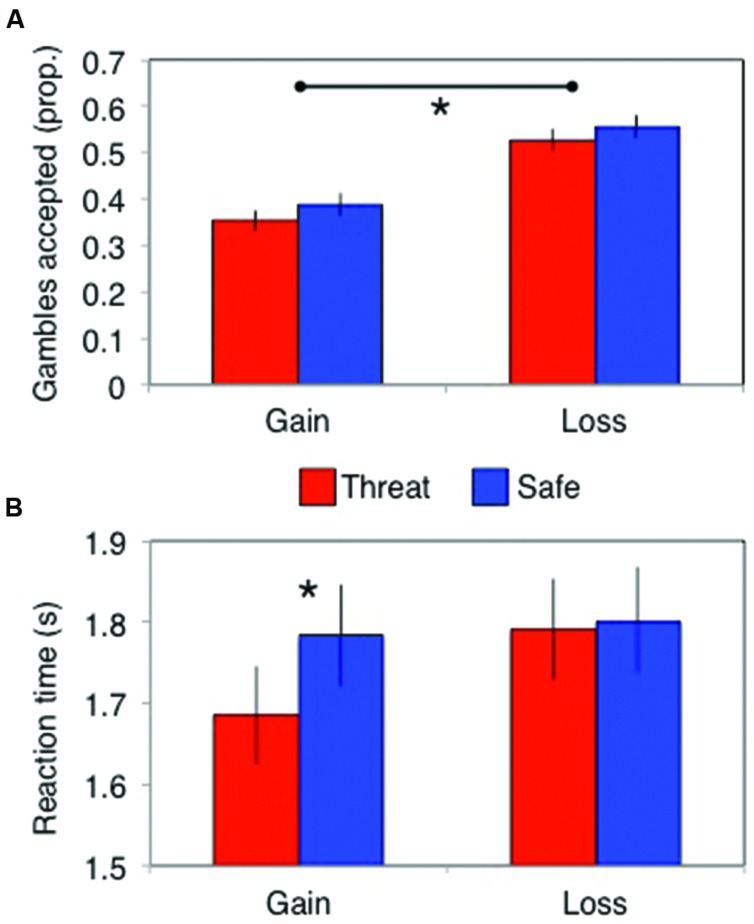
**(A)** The framing effect did not differ between stress conditions in terms of the proportion of gambles accepted, but **(B)** stress did speed up responses (in seconds) in the gains domain. Error bars indicate SEM; *p < 0.01.

A significant framing effect was demonstrated. Specifically, participants gambled more in the losses frame (probability of gambling = 0.54 ± 0.2) than in the gains frame [probability of gambling = 0.37 ± 0.2; main effect of frame: *F*(1,82) = 83, *p* < 0.001, $\eta_{p}^{2}$ = 0.5]. However, this did not interact with threat of shock [stress × frame interaction: *F*(1,82) = 0.13, *p* = 0.72, $\eta_{p}^{2}$ = 0.002; **Figure 2A**] and there was no main effect of stress [*F*(1,82) = 3.7, *p* = 0.06, $\eta_{p}^{2}$ = 0.043]. Bayes factor analysis revealed the winning model to be one including only a main effect of frame (logBF_10_ = 46), which was substantially (7.6 times) better than a model additionally including the stress × frame interaction (logBF_10_ = 44).

##### Reaction times

Subjects (only *N* = 74 had RTs in all cells, since some subjects never chose at least one of the options) were significantly faster to choose in the loss than gain frame [main effect of frame: *F*(1,74) = 7.5, *p* = 0.008, $\eta_{p}^{2}$ = 0.091] and under threat of shock [main effect of stress: *F*(1,74) = 5.0, *p* = 0.029, $\eta_{p}^{2}$ = 0.063]. These effects were qualified by a significant stress × frame interaction [*F*(1,74) = 7.0, *p* = 0.01, $\eta_{p}^{2}$ = 0.086]. Analyses of the simple main effects revealed that stress induced quicker responses in the gains domain [*F*(1,74) = 12, *p* = 0.001, $\eta_{p}^{2}$ = 0.14] but not in the losses domain [*F*(1,74) = 0.11, *p* = 0.74, $\eta_{p}^{2}$ < 0.002; **Figure 2B**]. Bayes factor analysis revealed that the winning model comprised individual main effects of stress and frame (logBF_10_ = 2.1), without the interaction; but this was only anecdotally (1.1 times, Jeffreys, 1998) better than the model also including a stress × frame interaction (logBF_10_ = 2.0).

#### (B) Between-Subjects Effects

##### Choice behavior

Neither safe-threat order [frame × order interaction: *F*(1,81) = 0.20, *p* = 0.66, $\eta_{p}^{2}$ = 0.002] nor baseline symptoms [frame × STAI interaction : *F*(1,81) = 1.4, *p* = 0.24, $\eta_{p}^{2}$ = 0.017; frame × BDI interaction: *F*(1,80) = 0.28, *p* = 0.60, $\eta_{p}^{2}$ = 0.003] interacted with any of the main effects of interest. There was no exploratory stress × frame × gender interaction [*F*(1,81) = 0.07, *p* = 0.8, $\eta_{p}^{2}$ = 0.001], stress × frame × age interaction [*F*(1,81) = 0.62, *p* = 0.53, $\eta_{p}^{2}$ = 0.008] or stress × frame × threat potentiated (threat minus safe) anxiety rating interaction [*F*(1,80) = 0.40, *p* = 0.53, $\eta_{p}^{2}$ = 0.005].

##### Reaction times

Neither safe–threat order [stress × frame × order interaction: *F*(1,73) = 0.20, *p* = 0.65, $\eta_{p}^{2}$ = 0.003] nor baseline symptoms [stress × frame × STAI interaction: *F*(1,73) = 0.11, *p* = 0.74, $\eta_{p}^{2}$ = 0.001; stress × frame × BDI interaction: *F*(1,72) = 1.3, *p* = 0.26, $\eta_{p}^{2}$ = 0.018] interacted with any of the interaction effects of interest.

### Temporal Discounting Task

#### (A) Within-Subjects Effects

##### Choice behavior

**FIGURE 3:**
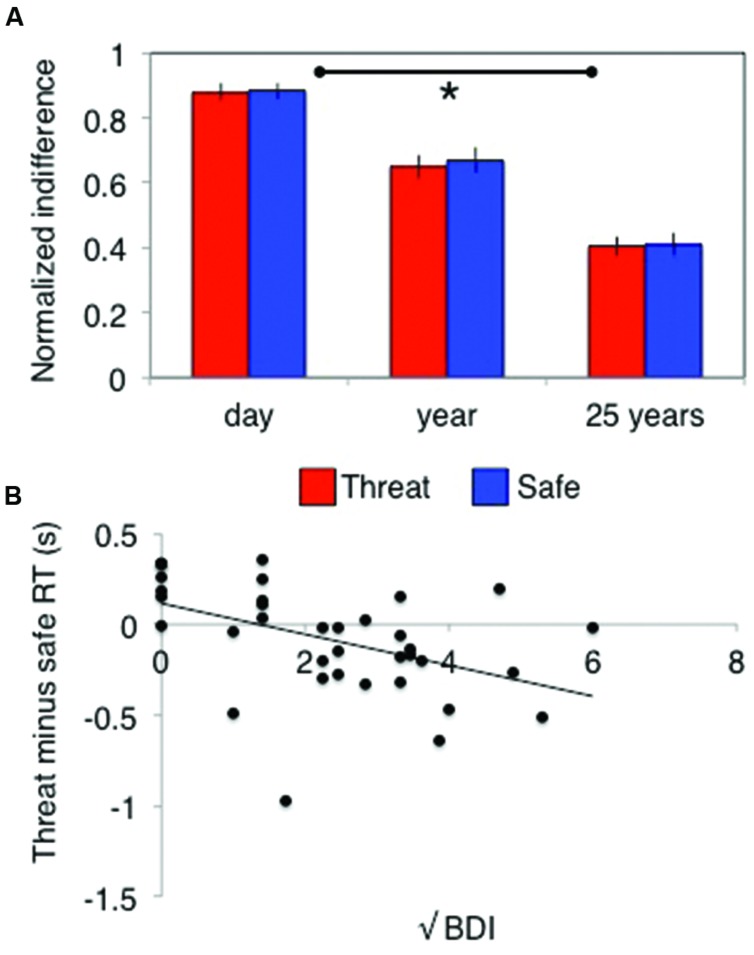
**Stress has **(A)** no behavioral effect on temporal discounting, but does **(B)** induce relative decision-speeding under threat in individuals with higher depressive symptoms.** Error bars indicate SEM; *p < 0.01.

Temporal discounting was demonstrated by a significant main effect of delay on indifference points [*F*(2,70) = 79, *p* < 0.001, $\eta_{p}^{2}$ = 0.7]. This varied depending upon whether subjects were asked about wins or losses [time × valence interaction: *F*(2,70) = 8, *p* = 0.001, $\eta_{p}^{2}$ = 0.2] but did not differ across the different values [time × value interaction: *F*(4,140) = 1.3, *p* = 0.26, $\eta_{p}^{2}$ = 0.04]. Critically, this also did not differ under stress [time × stress interaction: *F*(2,70) = 0.24, *p* = 0.79, $\eta_{p}^{2}$ = 0.007, **Figure 3A**; main effect of stress: *F*(1,35) = 0.8, *p* = 0.37, $\eta_{p}^{2}$ = 0.02; time × valence × stress: *F*(2,70) = 0.16, *p* = 0.86, $\eta_{p}^{2}$ = 0.004; time × valence × value × stress: *F*(4,140) = 0.73, *p* = 0.58, $\eta_{p}^{2}$ = 0.02]. Bayes factor analysis revealed a winning indifference point model comprising a time by valence interaction (logBF_10_ = 294) that was decisively (>150 times) better than model additionally including a stress by time interaction (logBF_10_ = 264), a stress by valence by time model (logBF_10_ = 271) or a time alone model (logBF_10_ = 271).

##### Reaction times

There was a main effect of time on RTs [*F*(2,70) = 9.9, *p* < 0.001, $\eta_{p}^{2}$ = 0.22], a main effect of valence [*F*(1,35) = 9.6, *p* = 0.004, $\eta_{p}^{2}$ = 0.22] and a significant valence × time interaction [*F*(2,70) = 7.9, *p* < 0.001, $\eta_{p}^{2}$ = 0.18]. Simple main effects analyses revealed that this interaction was driven by a main effect of time in the gain domain [subjects became progressively slower with increasing time: *F*(2,34) = 15, *p* < 0.001, $\eta_{p}^{2}$ = 0.47] but not the loss domain [subjects were always as slow as the slowest (i.e., 25 years) time point in the gains domain: *F*(2,34) = 1.8, *p* = 0.19, $\eta_{p}^{2}$ = 0.09]. There was no main effect of stress [*F*(1,35) = 2.6, *p* = 0.12, $\eta_{p}^{2}$ = 0.07] or stress by time interaction [*F*(2,70) = 0.8, *p* = 0.4, $\eta_{p}^{2}$ = 0.02]. There was a trend toward a stress × valence interaction [*F*(1,35) = 4.1, *p* = 0.050, $\eta_{p}^{2}$ = 0.11] but Bayes factor analysis revealed this model (logBF_10_ = 9) to be decisively (>150 times) worse than the winning valence × time model (logBF_10_ = 23).

#### (B) Between-Subject Effects

##### Choice behavior

No effects of interest interacted with task order [for indifference points, time × order interaction: *F*(2,68) = 1.1, *p* = 0.34, $\eta_{p}^{2}$ = 0.03] or baseline symptoms (for indifference points time × STAI interaction: *F*(2,68) = 0.51, *p* = 0.6, $\eta_{p}^{2}$ = 0.02; time × BDI interaction: *F*(2,68) = 0.27, *p* = 0.8, $\eta_{p}^{2}$ = 0.008]. There was no exploratory time × stress × gender interaction [*F*(2,68) = 0.26, *p* = 0.8, $\eta_{p}^{2}$ = 0.007], time × stress × age interaction [*F*(2,68) = 0.13, *p* = 0.88, $\eta_{p}^{2}$ = 0.004] or time × stress × threat potentiated (threat minus safe) anxiety rating interaction [*F*(2,66) = 1.4, *p* = 0.30, $\eta_{p}^{2}$ = 0.039]).

##### Reaction times

There was no time × order interaction [*F*(2,68) = 0.94, *p* = 0.4, $\eta_{p}^{2}$ = 0.03] or time × valence × order interaction [*F*(2,68) = 0.043, *p* = 0.96, $\eta_{p}^{2}$ = 0.001], but there was a significant stress × order interaction [*F*(1,34) = 16, *p* < 0.001, $\eta_{p}^{2}$ = 0.32] driven by those who experienced the safe condition first responding significantly faster under threat [*F*(1,34) = 17, *p* < 0.001, $\eta_{p}^{2}$ = 0.33; no difference between conditions in those who received threat first: *F*(1,34) = 2.5, *p* = 0.12, $\eta_{p}^{2}$ = 0.07]. There was no interaction between trait anxiety and any of the effects of interest (all *p*> 0.1), but there was a significant interaction between stress and BDI scores [*F*(1,34) = 9.1, *p* = 0.005, $\eta_{p}^{2}$ = 0.21] driven by a negative correlation [*r*(35) = –0.5] between the difference between threat and safe RTs and BDI (correlation substantially better model than null: logBF_10_ = 2.3; **Figure 3B**). In other words, the more depression symptoms an individual reported, the faster they responded under threat relative to safe conditions.

## Discussion

In this study, we were able to replicate two well-established biases in decision-making: the framing effect and temporal discounting. Moreover, we demonstrated a clear impact of threat of shock on subjective mood and choice reaction times. However, contrary to our predictions, stress did *not* alter the observed decision-making biases, perhaps because these executive decision-making biases are traits that are impervious to, or are able to override, the lower-level state affective biases induced by stress (Robinson et al., 2013c).

We first replicated the well-established framing effect, in which there is a bias toward risky behavior in the losses domain, and toward risk-aversion in the gains domain (Kahneman and Tversky, 1979). However, this bias was not altered by threat of shock in this study. To the best of our knowledge there is no previous literature exploring the impact of threat of shock on this effect, but there are a number of studies exploring the impact of different manipulations on framing. Using the cold pressor task Porcelli and Delgado (2009) found that the framing effect was enhanced by stress relative to a non-stress control condition. Given our sample size (*N* = 81), we had 99.1% statistical power (with alpha = 0.05; two-tailed) to replicate this interaction (with effect size |*d*| = 0.487; Porcelli and Delgado, 2009). One possible explanation for the discrepancy is simply that they used a different manipulation; very little, if any, work has directly compared threat of shock and cold pressor on stress responses (Robinson et al., 2013c). One key difference between paradigms, however, is that the cold pressor is generally completed *prior to* the task, since it requires the individual to submerge their hand in cold water. As such, it is plausible that it explores the impact of *recovery from* stress (Robinson et al., 2013c) rather than a current stressful context (which is a key advantage of the threat of shock technique used here). Another possibility highlighted by the authors is that their effect was a learning effect since the stress block always followed the no-stress block (Porcelli and Delgado, 2009).

Two further studies have explored the effects of another stress manipulation – the Trier social stressor test – on framing. Pabst et al found the *opposite* pattern to Porcelli and Delgado (2009); a *reduced* framing effect under stress (Pabst et al., 2013) albeit only in a subsample of their participants. Buckert et al. (2014) also showed *reduced* framing under social stress on a game of dice task. This discrepancy across manipulations could be attributed to the specific type of stress; whilst the cold pressor is physically painful, the Trier task asks subjects to ready themselves for unprepared public speaking which is a social form of stress. It is possible that the specific domain of anxiety influences the outcome; e.g., social rewards might be particularly influenced by a social anxiety induction. Nevertheless, adding our null effect with a further stressor to these inconsistent effects of stress leaves the role of stress on framing unclear (**Table 1**). This lack of clarity also highlights the need for work directly comparing different stress manipulations across the same tasks.

**Table 1:** Review of findings cited in this paper (=, null effect; N/A, not cited; ↑, increased effect; ↓, reduced effect).

	Framing	Temporal discounting
Cold pressor	↑	N/A
Social stress	↓	=
Threat of shock	=	=

In our second experiment we replicated the temporal discounting effect. Specifically, participants assigned less utility to outcomes in the future. Again, however, we failed to detect an impact of stress on this bias. Temporal discounting has not been assessed under threat of shock to our knowledge, but this null finding is consistent with at least three prior studies (Lempert et al., 2012; Haushofer et al., 2013; Jenks and Lawyer, 2015) utilizing the Trier social stressor test. In all three studies stress increased both subjective and/or hormonal indicators of stress, but had no effect on discounting (**Table 1**). Where anxiety *has* been associated with temporal discounting it is in studies exploring *between-subject* individual differences in social anxiety (Rounds et al., 2007) or clinically defined depression (Pulcu et al., 2014). In both cases this is consistent with temporal discounting being a stable, *trait* measure (Odum, 2011). Temporary or acute mood fluctuations such as stress or anxiety may be therefore be unable to influence such traits. That said, it should be noted that we fail to see an interaction with trait depression or anxiety symptoms in our sample and the Rounds effect cited above recently failed to replicate (Jenks and Lawyer, 2015) so firm conclusions are perhaps unwarranted.

We do, however, see some evidence of stress impacting reaction time across both tasks. This is critical because it suggests that the threat of shock manipulation was effective at instantiating behavioral change. In other words, in addition to the subjective reports of anxiety, subjects’ responses *were* impacted by threat, even though these did not carry over into their decisions. Reaction time effects in the absence of decision effects are consistent with prior work (Murphy, 1959; Keinan, 1987; Engelmann et al., 2015) and perhaps reflect a bias toward making some decisions faster under conditions of stress. From an evolutionary perspective, such a mechanism could be adaptive: a faster decision about which direction to go when running away from a predator, for instance, may improve survival chances. It should be noted, moreover, that the reaction time correlation with depression symptoms we see in the temporal discounting reaction time effect, somewhat mimics our previously reported effect in the Iowa Gambling Task (Robinson et al., 2015). In that study, we saw increased selection of disadvantageous decks under threat relative to safe in individuals with high anxiety or depression symptomatology (Robinson et al., 2015). Here we observed (in a partially overlapping sample) quicker responses under threat relative to safe conditions in those who reported greater depressive symptoms. It is plausible that these effects may represent some form of underlying vulnerability in individuals with subclinical depressive symptoms. Having said that, the effect in the present task was not also observed in an relationship with trait anxiety scores (unlike our prior report), which is surprising because trait anxiety and BDI scores are highly correlated in most samples (including this one: *R* = 0.8, *p* < 0.001). Whilst it is possible that the present effect is specific to depression vs. anxiety symptoms we feel that this conclusion would be unwarranted based on the current data, and it requires replication in a larger sample. Reaction time effects can, however, have multiple underlying causes; the effects could be driven by altered decision-making processes, but could also be driven by the time it takes to encode or instantiate a reaction toward a stimulus (Ratcliff and McKoon, 2008) and it is not possible to fully distinguish between these possibilities. Recent work has in fact highlighted the need for researchers to be extremely cautious when using reverse-inference to infer cognitive states from reaction time (Krajbich et al., 2015). Indeed, in general, the exact nature of the reaction time effects seen here were not predicted *a priori*, and in one instance the Bayesian and frequentist tests are partially discrepant (within-subject temporal discounting p = 0.050 vs. 150 times worse) and as such we do not wish to draw firm conclusions beyond observing that the effects indicate that the manipulation was having some effect during the tasks.

This raises the question as to why is there are clear affective biases under threat of shock (Robinson et al., 2013c), but that these biases do not impact decisions. One speculation is that this is because such ‘bottom–up’ biases do not influence some higher level, executive processes. Or, if the biases *are* processed later in the hierarchy, it may be that the executive *overrides* lower level biases. Evolutionarily, an ability of executive function to ignore or override lower level fear and stress responses might be adaptive in certain circumstances. Alternatively, these biases might reflect the use of highly efficient heuristics/rules of thumb which are robust to the effects of stress on affective processing. Lower level affective biases may therefore constitute *state* effects of mood disorders that change with symptoms, whilst the executive decision-making biases constitute stable *traits*. Such traits may contribute to stress-related disorder susceptibility (Pulcu et al., 2014), but not change with mood symptoms. Understanding the distinction between different levels of cognitive function that are impacted by stress might plausibly inform our treatments for stress-related disorders. Specifically, the focus might be on shifting lower-level *state* affective biases rather than *trait* executive biases. Either way, in contrast to our hypotheses the present study provides evidence for the proposition that certain higher order executive decision-making functions are impervious to stress induced by threat of shock.

### Limitations

It should be noted that one explanation for our lack of effect is that our stress manipulation was not strong enough to elicit change. Perhaps under conditions of extreme threat (e.g., a warzone or high-stakes work environment) decision-making of this type can be shifted by threat. Alternatively given higher time pressure, higher financial gambles, or explicit feedback about the outcomes of gambles, individual’s decisions would have been shifted by threat of shock. Moreover, as discussed above, there are many different ‘stress’ manipulations across social, pain, and other domains and it is unclear exactly how these overlap. The extent to which this non-significant effect of stress generalizes across stress manipulations is unclear. In addition, these findings do not rule out the impact of threat of shock on other types of decision-making such as those that involves working memory or inhibitory control [both of which have in fact been shown to be influenced by threat (Robinson et al., 2013a,c)]. Overall, a non-significant effect of this nature is difficult to prove as it may simply be that we have failed to find the correct context in which stress impairs the executive functions explored here. A further limitation is that these individuals were not screened using a diagnostic interview. As such, some individuals may have previous diagnoses which they had forgotten, or may have been missed by using a checklist screening instrument. Finally, these findings do not of course rule out the possibility that positive mood might have effects on these sorts of decision making tasks. Indeed there is preliminary (albeit complex) data suggesting that positive mood can influence temporal discounting (Hirsh et al., 2010) and Iowa Gambling Task performance (De Vries et al., 2008).

## Conflict of Interest Statement

The authors declare that the research was conducted in the absence of any commercial or financial relationships that could be construed as a potential conflict of interest.

## Footnotes

1 Note that the ‘BF_10_’ nomenclature in JASP refers to the Bayes factor for H1 vs. H0 (model relative to null); as distinct from ‘BF_01_,’ which is the Bayes factor for H0 vs. H1 (null relative to model). It does *not* refer to log_10._ Where we refer to ‘logBF_10_,’ we report the natural log of the BF_10_ values. This log is not required; it is simply used to make the frequently very large numbers more interpretable.

2 http://dx.doi.org/10.6084/m9.figshare.1423293
